# Circ_0001741 regulates proliferation and invasion in ESCC via the miR-194-5p/E2F3 axis

**DOI:** 10.1186/s12957-025-04124-2

**Published:** 2025-12-10

**Authors:** Yuanyuan Wang, Qianqian Sun, Zhuyao Li

**Affiliations:** 1 Clinical Systems Biology Laboratories, The First Affiliated Hospital of Zhengzhou University, Zhengzhou, 450001 China; 2 Institute of Infection and Immunity, Henan Academy of Innovations in Medical Science, Zhengzhou, China; 3 Zhengzhou Key Laboratory of Children’s Infection and Immunity, Zhengzhou Children’s Hospital, Zhengzhou, China; 4 Department of Thyroid Surgery, The First Affiliated Hospital of Zhengzhou University, Zhengzhou, China

**Keywords:** Esophageal squamous cell carcinoma (ESCC), Circ_0001741, E2F3, Invasion, MiR-194-5p

## Abstract

**Purpose:**

Esophageal squamous cell carcinoma (ESCC) is a highly aggressive gastrointestinal malignancy. This study aims to investigate the role and molecular mechanism of a novel circular RNA, circ_0001741, in ESCC progression.

**Methods:**

The circular structure of circ_0001741 was confirmed by RNase R and actinomycin D assays alongside divergent primer-based amplification targeting its backsplice junction (BSJ). Its expression was profiled using an ESCC tissue microarray. Functional effects of circ_0001741 on proliferation and invasion were assessed using CCK-8 and Transwell assays. The interactions within the circ_0001741/miR-194-5p/E2F3 axis were validated through dual-luciferase reporter, RNA pull-down, and rescue experiments.

**Results:**

Circ_0001741 was significantly upregulated in ESCC tissues with a 7.5-fold increase compared to adjacent normal tissues. Functional assays demonstrated that silencing circ_0001741 markedly inhibited ESCC cell proliferation and invasion significantly. Mechanistically, circ_0001741 directly sponged miR-194-5p, leading to the upregulation of its target oncogene E2F3. Crucially, the anti-tumor effects induced by circ_0001741 knockdown were significantly reversed by co-silencing miR-194-5p or overexpressing E2F3, thus establishing a functional circ_0001741/miR-194-5p/E2F3 axis in ESCC.

**Conclusion:**

Our findings establish that circ_0001741 drives ESCC progression by modulating the miR-194-5p/E2F3 axis, underscoring its therapeutic potential for ESCC treatment.

**Supplementary Information:**

The online version contains supplementary material available at 10.1186/s12957-025-04124-2.

## Introduction

Esophageal cancer ranks among the most prevalent malignancies of the digestive tract worldwide, accounting for nearly 600,000 annual fatalities [1–4]. Patients diagnosed with esophageal squamous cell carcinoma (ESCC) face particularly poor prognoses due to aggressive local invasion and distant metastases, resulting in substantially reduced survival rates [5]. While early-stage non-metastatic ESCC cases may achieve remission or cure through intervention [6], metastatic disease carries a dismal prognosis with a 5-year survival rate of merely 3.4% [7]. This stark contrast underscores the critical need to elucidate molecular mechanisms driving ESCC invasion, which could enable earlier detection and therapeutic interventions to improve patient outcomes.

Recent advances in high-throughput sequencing have revealed numerous non-coding RNAs with significant implications in carcinogenesis and disease progression [8, 9]. Circular RNAs (circRNAs), a conserved subclass of non-coding RNAs, function as molecular sponges that sequester microRNAs (miRNAs) through competitive binding, thereby modulating gene expression and influencing critical cellular processes [10–14]. Dysregulation of circRNA expression has been implicated in various pathological conditions, including multiple cancer types [10, 15–19]. Specifically in ESCC, recent studies have continued to uncover novel functional circRNAs and their mechanisms [20]. For instance, emerging research has identified that a novel protein encoded by circUBE4B promotes ESCC progression by augmenting MAPK/ERK signaling, highlighting the protein-coding potential of circRNAs as a critical oncogenic mechanism [21]. This aligns with the broader prediction that circRNAs are poised to lead to revolutionary breakthroughs in cancer diagnosis and therapy within the next 5–10 years [22].

In our preliminary analysis of ESCC tissues, circ_0001741 emerged as a novel circRNA with aberrantly elevated expression compared to adjacent normal tissues. While the importance of circRNAs in ESCC is increasingly recognized, the specific functional role and molecular mechanism of circ_0001741 in ESCC pathogenesis remain unclear. This study therefore aims to systematically investigate the biological role of circ_0001741 and mechanistically delineate its potential tumor-promoting effects. We hypothesize that circ_0001741 functions as an oncogenic circRNA in ESCC. Our findings are anticipated to provide new insights into the regulatory network of circRNAs in ESCC progression and may suggest novel potential therapeutic targets.

## Methods

### Tissue microarray

A tissue microarrays containing human ESCC and paired adjacent non-cancerous tissues was procured from Outdo Biotech Co., Ltd. (Shanghai, China).

### Circular RNA validation

To confirm the circular structure of circ_0001741, divergent primers were designed to amplify its back-splice junction (BSJ), based on its theoretical mature sequence derived from exons 2–4 of the host gene TNPO3. The PCR products were sequenced and analyzed using BLAST to verify the circular nature.

### Quantitative real-time PCR

Total RNA was extracted using TRIzol reagent (Invitrogen, USA). cDNA was synthesized using the PrimeScript RT reagent Kit (Takara, Iapan, Cat No. RR037B). Quantitative real-time PCR (qRT-PCR) was performed using the TB Green Premix Ex Taq II kit (Takara, Iapan, Cat No. RR820B). The reaction conditions were as follows: 95 ℃ for 30 s, followed by 40 cycles of 95 ℃ for 5 s and 60 ℃ for 30 s. GAPDH was used as an internal control for mRNA and circRNA, and U6 snRNA was used for miRNA. Three replicate wells were prepared for each gene. The relative expression levels were quantified using the threshold cycle (Ct) method and calculated using the 2^−ΔΔCt^ method.

### RNase R and actinomycin D treatment

Total RNA (3 µg) was incubated with 9 units of RNase R (Geneseed Biotech, China) for 15 min at 37 °C. circ_0001741 and E2F mRNA expression levels were subsequently analyzed by qRT-PCR. To inhibit transcription, cells were treated with 2 µg/mL actinomycin D (Sigma-Aldrich, USA) for 0, 2, 4, 8, 16, and 24 h. Total RNA was then extracted, and circ_0001741/E2F mRNA stability was assessed via qRT-PCR.

### Cell culture

Human ESCC cell lines (KYSE150 and TE2) were acquired from the Type Culture Collection of the Chinese Academy of Sciences (Shanghai, China). KYSE150 and TE2 cells were cultured in RPMI-1640 medium (Gibco, USA) supplemented with 10% fetal bovine serum (FBS, Gibco, USA) and 1% penicillin-streptomycin (Gibco, USA). The normal esophageal epithelial cell line (HET-1 A) was cultured in DMEM medium (Gibco, USA) supplemented with 10% FBS and 1% penicillin-streptomycin. All cells were maintained in a humidified incubator at 37 °C with 5% CO_2_.

### Cell transfection

Transfection was performed using Lipofectamine 3000 reagent (Invitrogen, USA) according to the manufacturer’s instructions. Briefly, for a 6-well plate, 1.5 µg of plasmid DNA or 50 nM of siRNA/miRNA mimic/inhibitor was diluted in 125 µL of Opti-MEM Reduced Serum Medium. Separately, 3.75 µL of Lipofectamine 3000 reagent was diluted in 125 µL of Opti-MEM. The diluted DNA/RNA was combined with the diluted Lipofectamine 3000 reagent, incubated for 15 min at room temperature to form complexes, and then added dropwise to the cells. The culture medium was replaced with fresh complete medium 6 h post-transfection. siRNAs targeting circ_0001741 (si-circ_0001741), scrambled siRNA (si-NC), wild-type circ_0001741 plasmid (WT-circ_0001741), mutant circ_0001741 plasmid (Mut-circ_0001741), miR-194-5p mimic, and negative control miRNA (miR-NC) were synthesized by GenePharma (Shanghai, China).

### CCK-8 assay

Cell viability was evaluated daily for four consecutive days using the Cell Counting Kit-8 (CCK-8; Dojindo Laboratories, Japan). Absorbance at 450 nm (OD_450_) was measured to generate cell survival curves.

### Transwell assay

Cell invasion was assessed using Transwell chambers (8 μm pore size; Corning, USA) pre-coated with Matrigel (Corning, USA). Briefly, 5 × 10^4^ cells resuspended in 200 µL of serum-free medium were seeded into the upper chamber. The lower chamber was filled with 600 µL of medium containing 15% FBS as a chemoattractant. After 24 h of incubation, the non-invading cells on the upper surface of the membrane were removed with a cotton swab. The invaded cells on the lower surface were fixed with 4% paraformaldehyde, stained with 0.1% crystal violet, and photographed and counted under a light microscope. Each experiment was performed with three technical replicates and repeated three times independently.

### Dual-luciferase assay

DNA fragments encompassing wild-type or mutant miR-194-5p binding sites in the E2F3 3’UTR or circ_0001741 sequence was cloned into the pmirGLO dual-luciferase reporter vector (Promega, USA). Cells were co-transfected with miRNA mimics and reporter plasmids. Luciferase activity was measured 48 h post-transfection using the Dual-Luciferase Assay System (Promega, USA), with firefly luciferase normalized to Renilla luciferase.

### Western blotting

Total protein was extracted using RIPA lysis buffer (Beyotime, China) containing 1 mM PMSF (Beyotime, China). Protein concentrations were determined using a BCA Protein Assay Kit (Beyotime, China). Equal amounts of proteins were separated by SDS-PAGE, transferred to PVDF membranes (Millipore, USA), and blocked with 5% non-fat milk. Membranes were incubated overnight at 4 °C with E2F3 primary antibodies (Proteintech, China, Cat No. 27615-1-AP), followed by HRP-conjugated secondary antibodies (Proteintech, China). GAPDH (Proteintech, China, Cat No. 10494-1-AP) served as a loading control.

#### RNA Pull-down

Biotinylated RNA probes (Bio-circ_0001741-wt/mut and Bio-E2F3-wt/mut) were hybridized with KYSE150/TE2 cell lysates for 4 h. RNA-protein complexes were captured with streptavidin-coated magnetic beads (Thermo Fisher Scientific, USA). Following RNA extraction, miR-194-5p abundance in the bound fraction was quantified via qRT-PCR.

### Statistical analysis

Statistical analysis was conducted using SPSS 21.0 software. Data are expressed as mean ± standard deviation (SD). Intergroup comparisons were analyzed using one-way ANOVA and Student’s *t*-test, with statistical significance defined as *P* < 0.05.

## Results

### Characteristics of circ_0001741 in ESCC

**Fig. 1:**
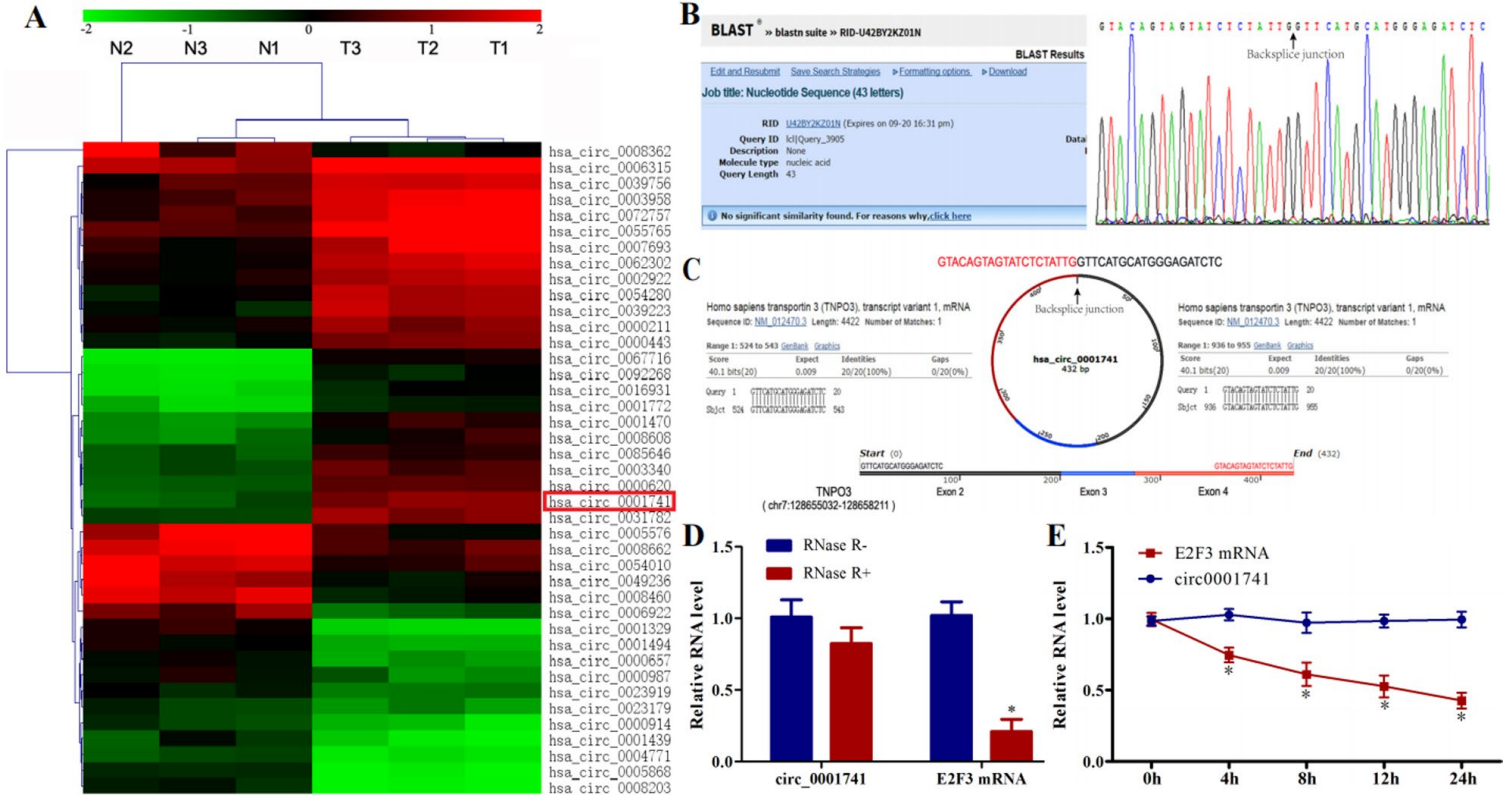
circ_0001741 is a circular RNA in ESCC **A** CircRNA expression profiles in ESCC and paired normal tissues **B** BLAST analysis of the BSJ region **C** Schematic of circ_0001741 formation from *TNPO3* exons 2–4 **D** qRT-PCR analysis of circ_0001741 and *TNPO3* mRNA after RNase R treatment **E** Stability of circ_0001741 and *TNPO3* mRNA following Actinomycin D treatment. *P < 0.05

Our previous high-throughput circRNA sequencing revealed significant upregulation of circ_0001741 in ESCC tissues compared to adjacent normal tissues (7.5-fold increase, *P* < 0.05; Fig. 1A). Sequencing and BLAST analysis of the BSJ region revealed that the sequence upstream of the BSJ corresponded to exon 4 of the *TNPO3* gene, while the downstream sequence aligned with exons 2 and 3 of *TNPO3* (Fig. 1C). No significant matches were detected for the BSJ region itself (Fig. 1B), confirming the circular nature of circ_0001741.

To validate its circular nature, we confirmed its resistance to RNase R digestion and its stability upon Actinomycin D treatment. RNase R treatment significantly degraded linear *TNPO3* mRNA (*P* < 0.05) but had no effect on circ_0001741 levels (Fig. 1D). Similarly, Actinomycin D reduced *TNPO3* mRNA expression while leaving circ_0001741 largely unaffected (*P* < 0.05, Fig. 1E). These findings collectively confirm the circular structure and stability of circ_0001741.

### Downregulation of circ_0001741 inhibits ESCC cell proliferation and invasive

**Fig. 2:**
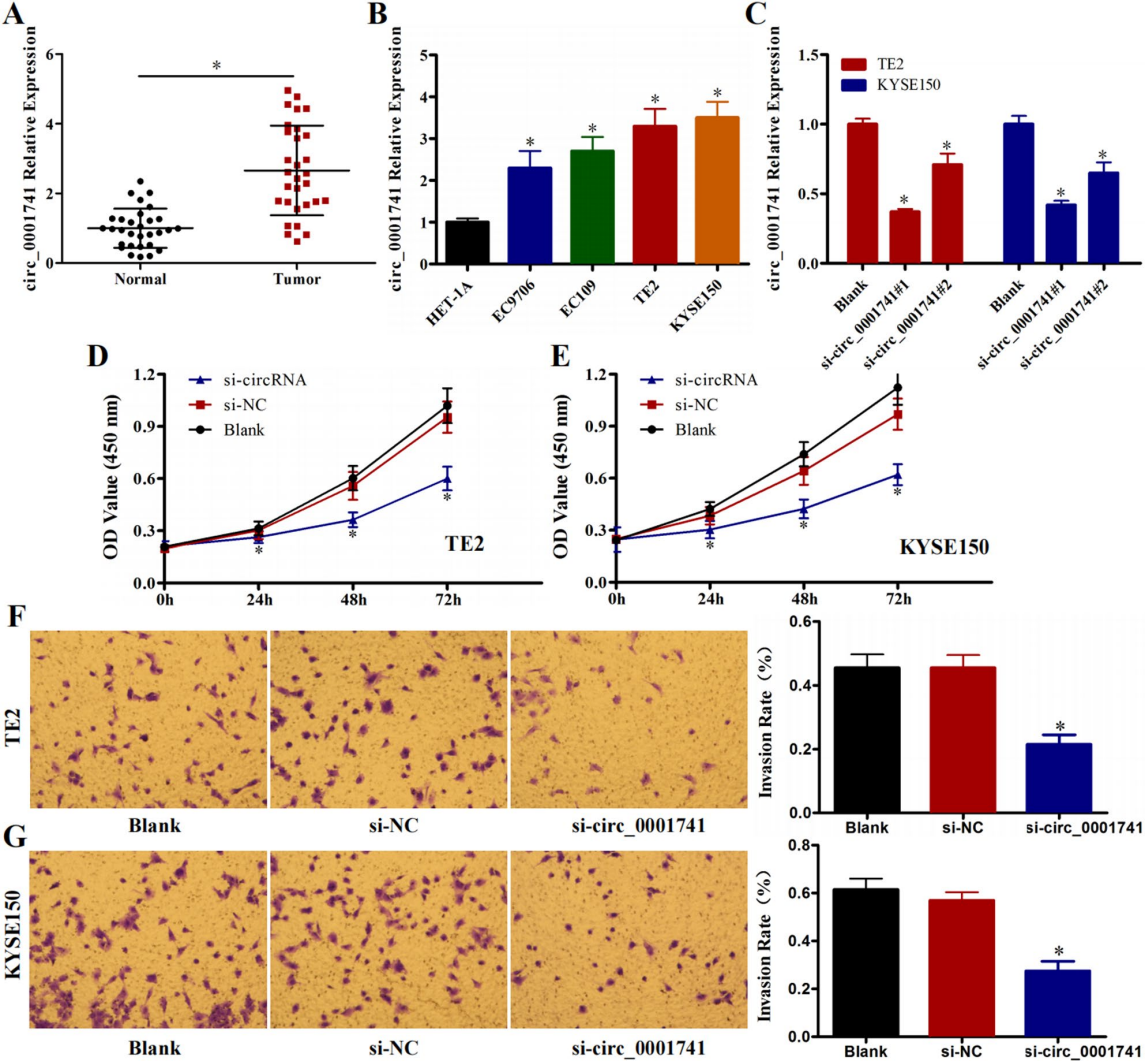
circ_0001741 downregulation inhibits ESCC cell proliferation and invasive **A** circ_0001741 expression is elevated in 30 ESCC tissues compared to paired adjacent normal tissues **B** circ_0001741 expression in ESCC cell lines EC9706, EC109, TE2, KYSE150 and normal esophageal epithelial cell line HET-1 A **C** circ_0001741 expression in ESCC cells transfected with si-circ_0001741. (**D**, **E**) CCK-8 assays assessing the effect of circ_0001741 knockdown on proliferation in TE2 and KYSE150 cells. (**F**, **G**) Transwell assays evaluating the impact of circ_0001741 knockdown on invasion in TE2 and KYSE150 cells. *P < 0.05

Furthermore, expression analysis using a tissue microarray confirmed the elevated expression level of circ_0001741 in clinical ESCC samples (*P* < 0.05, Fig. 2A). qRT-PCR quantification in ESCC cell lines EC9706, EC109, TE2, KYSE150 and normal esophageal epithelial cells HET-1 A revealed highest circ_0001741 expression in TE2 and KYSE150 cells (*P* < 0.05, Fig. 2B). Transfection with si-circ_0001741^#1^ (selected for optimal silencing efficiency, *P* < 0.05, Fig. 2C) significantly reduced circ_0001741 levels. CCK-8 assays demonstrated that circ_0001741 knockdown suppressed proliferation in TE2 and KYSE150 cells compared to si-NC and Blank controls (*P* < 0.05, Fig. 2D and E). Similarly, invasion assays showed fewer invasive cells in the si-circ_0001741 group (*P* < 0.05, Fig. 2F and G), indicating that circ_0001741 downregulation inhibits ESCC cell proliferation and invasive in vitro.

### miR-194-5p is a direct target of circ_0001741 in ESCC cells

**Fig. 3:**
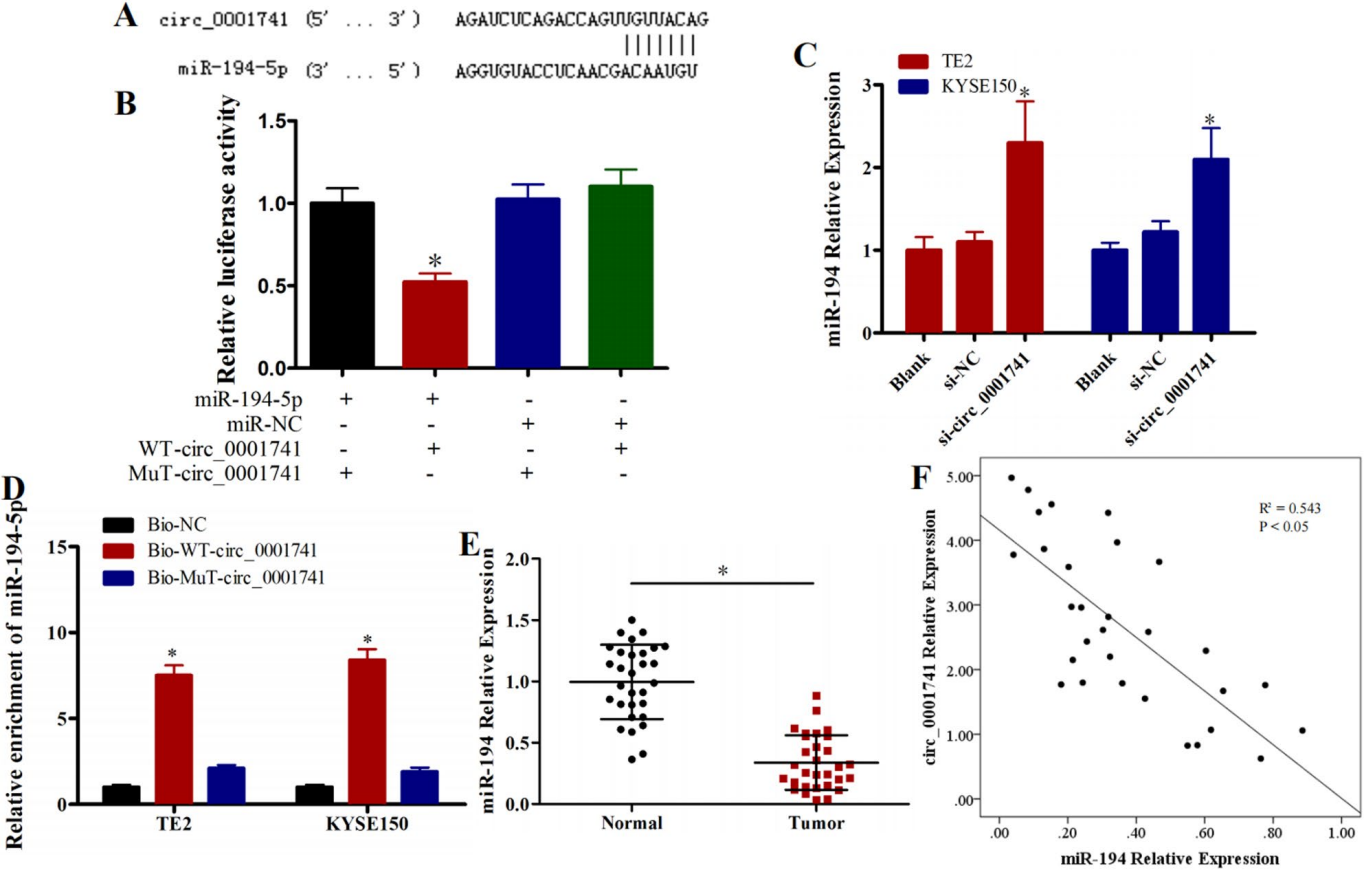
miR-194-5p is a target of circ_0001741 in ESCC cells **A** Predicted partially complementary binding sequence between circ_0001741 and miR-194-5p **B** Dual-luciferase reporter assays confirming circ_0001741–miR-194-5p interaction **C** Pull-down assays validating circ_0001741–miR-194-5p binding **D** qRT-PCR analysis of miR-194-5p expression in TE2 and KYSE150 cells after circ_0001741 silencing **E** miR-194-5p expression levels in 30 ESCC tissues and paired normal tissues **F** Correlation between circ_0001741 and miR-194-5p expression in 30 ESCC tissues. *P < 0.05

To elucidate the mechanism underlying circ_0001741-mediated ESCC invasive, we employed three bioinformatics platforms circBank, starBase, and circinteractome to identify potential miRNA targets of circ_0001741. This systematic analysis identified miR-194-5p as a strong candidate interacting with circ_0001741. Figure 3A illustrates the partially complementary binding sequence between circ_0001741 and miR-194-5p. To experimentally validate the predicted interaction between circ_0001741 and miR-194-5p, we performed dual-luciferase reporter assays. We first confirmed the transfection efficiency of the nucleic acid constructs to ensure the reliability of the assay (*P* < 0.05, Fig. S1). Subsequent analysis revealed a significant reduction in luciferase activity in cells co-transfected with the wild-type circ_0001741 reporter plasmid (WT-circ_0001741) and the miR-194-5p mimic, compared to the respective control groups (*P* < 0.05, Fig. 3B). In contrast, luciferase activity remained unchanged in cells transfected with the mutant circ_0001741 reporter (Mut-circ_0001741), irrespective of whether the miR-194-5p mimic was present or not (Fig. 3B). RNA pull-down assays further confirmed specific enrichment of miR-194-5p by WT-circ_0001741, but not Mut-circ_0001741 (*P* < 0.05, Fig. 3C).

qRT-PCR analysis revealed that circ_0001741 silencing significantly upregulated miR-194-5p expression in both TE2 and KYSE150 cell lines (*P* < 0.05, Fig. 3D). correlation analysis showed markedly reduced miR-194-5p levels in 30 ESCC tissue samples compared to matched normal tissues (*P* < 0.05, Fig. 3E), with a significant inverse correlation between circ_0001741 and miR-194-5p expression (R^2^ = 0.543, *P* < 0.05, Fig. 3F). Collectively, these findings establish miR-194-5p as a direct downstream target of circ_0001741 in ESCC cells, where circ_0001741 downregulation elevates miR-194-5p expression levels.

### miR-194-5p reverses circ_0001741 knockdown effects

**Fig. 4:**
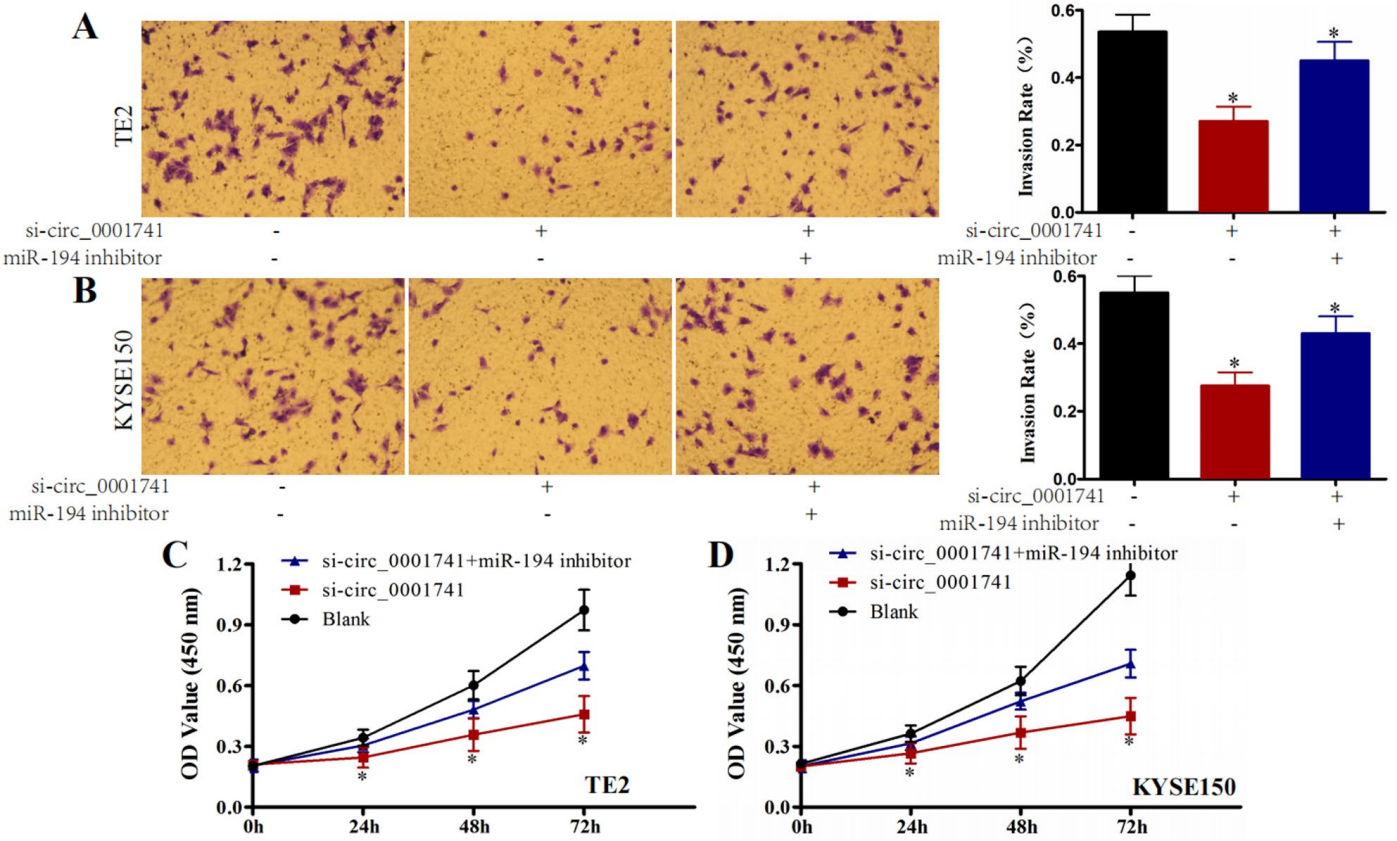
miR-194-5p silencing reverses the effects of circ_0001741 knockdown in TE2 and KYSE150 cells. (**A**, **B**) Transwell assays showing that miR-194-5p inhibition rescues the anti-invasive effects of circ_0001741 knockdown. (**C**, **D**) CCK-8 assays demonstrating that miR-194-5p silencing restores proliferation suppressed by circ_0001741 knockdown. *P < 0.05

To investigate whether circ_0001741 regulates E2F3 through miR-194-5p, we examined whether miR-194-5p inhibition could rescue the metastatic effects of circ_0001741 knockdown. Cells were divided into three experimental groups: Blank control, si-circ_0001741, and si-circ_0001741 + miR-194-5p inhibitor. Transwell assays revealed that circ_0001741 silencing significantly reduced cellular invasion in TE2 and KYSE150 cells (*P* < 0.05, Fig. 4A and B). This anti-invasive effect was partially reversed by miR-194-5p co-silencing. Similarly, CCK-8 assays demonstrated reduced proliferation in circ_0001741-deficient cells, which was attenuated by miR-194-5p inhibition (*P* < 0.05, Fig. 4C and D). These results confirm that circ_0001741 knockdown suppresses oncogenic behaviors in esophageal cancer cells through miR-194-5p-mediated mechanisms.

### E2F3 is a miR-194-5p target

**Fig. 5:**
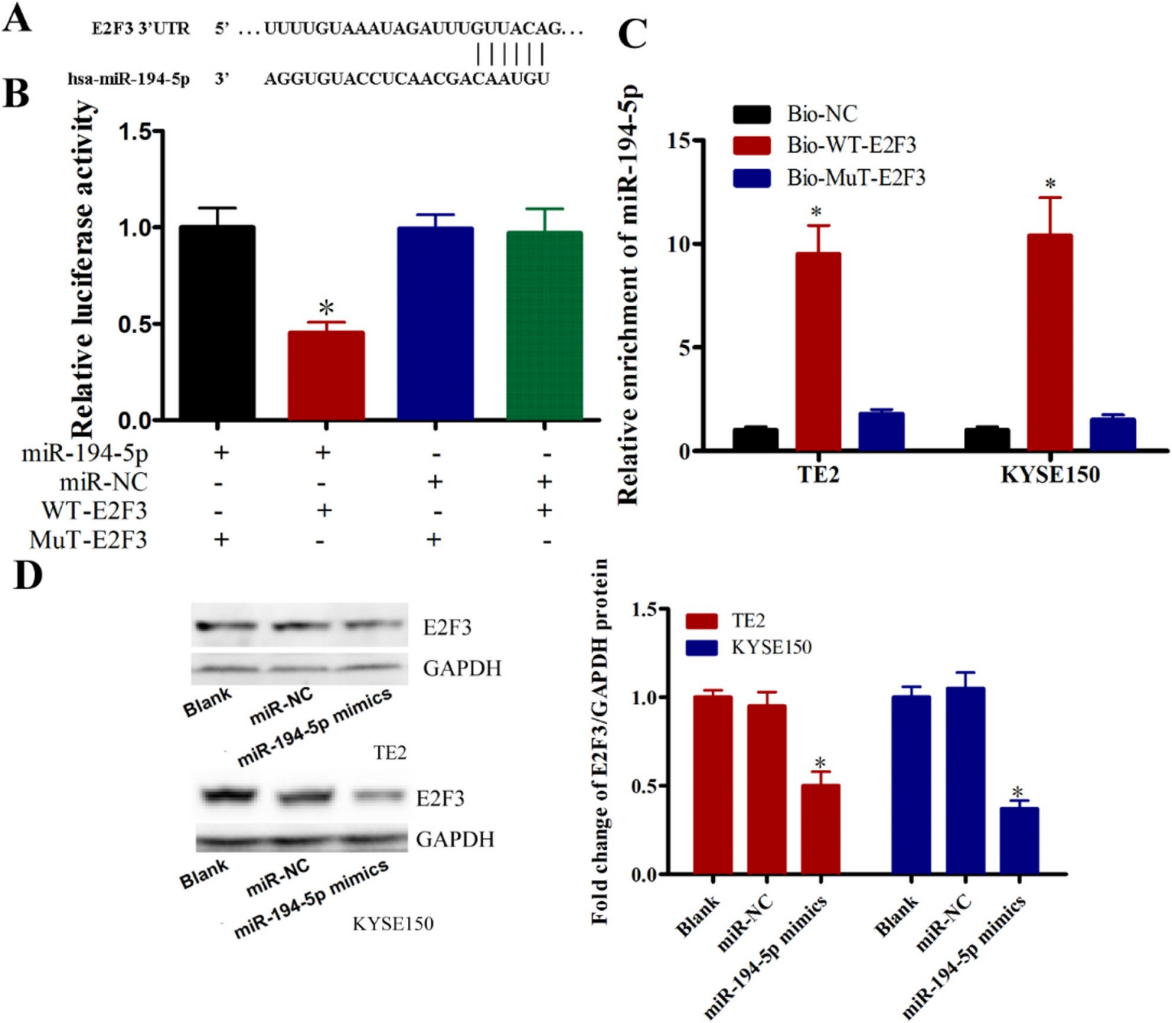
E2F3 is a miR-194-5p target gene in TE2 and KYSE150 cells **A** Predicted partially complementary binding sequence between E2F3 mRNA and miR-194-5p **B** Dual-luciferase reporter assays confirming miR-194-5p binding to the E2F3 3’UTR. **C** Pull-down assay was used to confirm the interaction of E2F3 and miR-194-5p **D** Western blot analysis of E2F3 protein levels in TE2 cells after miR-194-5p overexpression. *P < 0.05

Bioinformatic analysis identified E2F3 as a putative miR-194-5p target, with complementary binding sequences predicted (Fig. 5A). Dual-luciferase assays showed significantly reduced activity in cells co-transfected with WT-E2F3 3’UTR and miR-194-5p mimic compared to controls (*P* < 0.05, Fig. 5B). Mutant E2F3 constructs showed no response to miR-194-5p manipulation. RNA pull-down assays confirmed direct interaction between miR-194-5p and WT-E2F3 (*P* < 0.05, Fig. 5C). Western blot analysis revealed miR-194-5p overexpression significantly reduced E2F3 protein levels in both cell lines compared to controls (*P* < 0.05, Fig. 5D). Collectively, these findings establish miR-194-5p as a direct regulator of E2F3 expression.

### E2F3 rescues circ_0001741/miR-194-5p effects

**Fig. 6:**
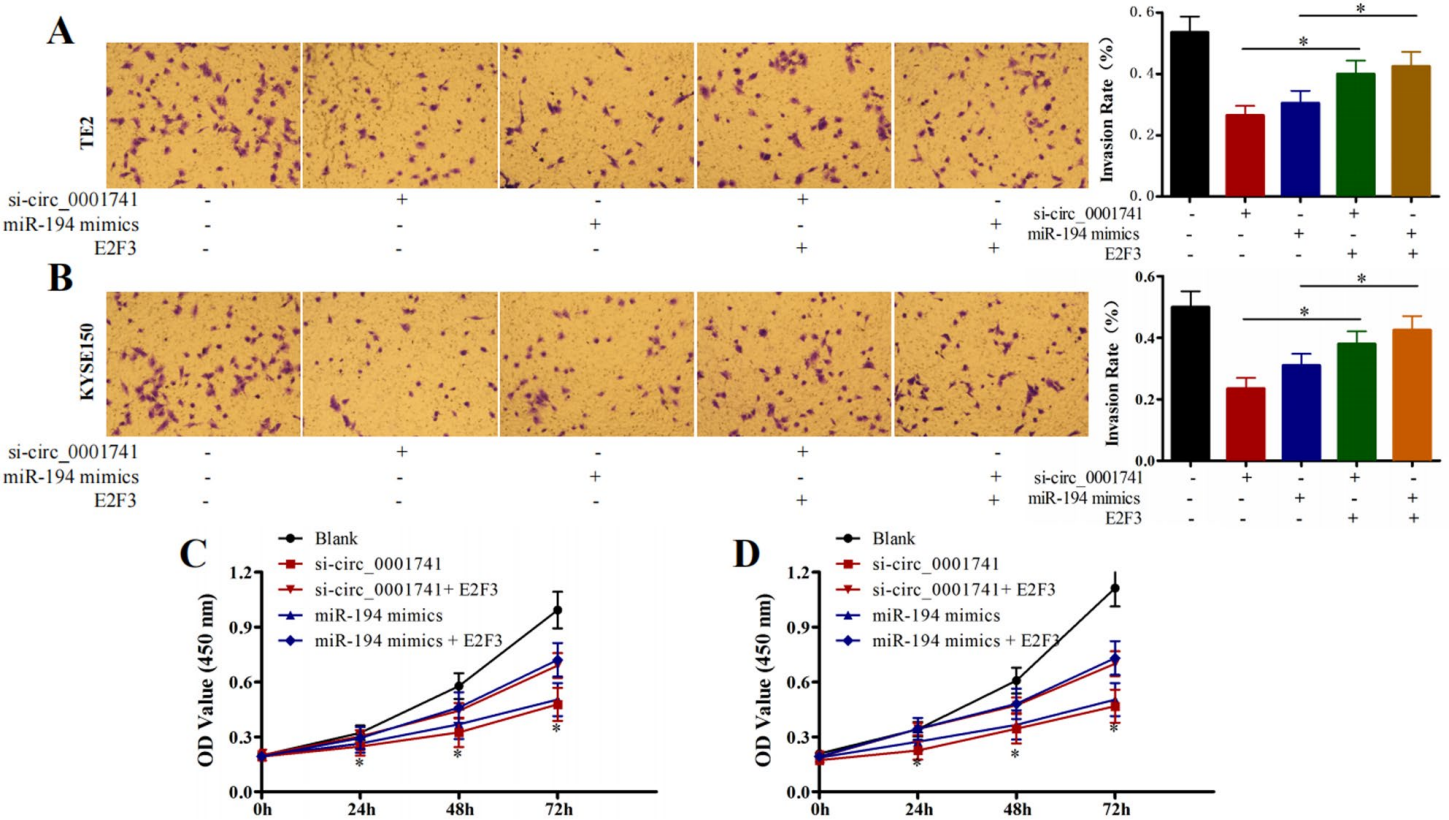
E2F3 overexpression reverses the effects of circ_0001741 knockdown **A** E2F3 overexpression partially rescues the anti-invasive effects of si-circ_0001741 or miR-194-5p in TE2 and KYSE150 cells **B** E2F3 overexpression partially restores proliferation suppressed by si-circ_0001741 or miR-194-5p. *P < 0.05

To investigate whether E2F3 overexpression could counteract the tumor-suppressive effects of circ_0001741 silencing, we performed rescue experiments using five experimental groups: Blank control; si-circ_0001741; miR-194-5p mimic; si-circ_0001741 + E2F3 overexpression; miR-194-5p mimic + E2F3 overexpression. Both circ_0001741 knockdown and miR-194-5p mimic significantly reduced cellular invasion (*P* < 0.05, Fig. 6A and B) and proliferation (*P* < 0.05, Fig. 6C and D) in TE2 and KYSE150 cells. Notably, E2F3 overexpression substantially attenuated these inhibitory effects on invasion capacity and proliferative activity. These results demonstrate that circ_0001741 silencing suppresses esophageal cancer cell progression through miR-194-5p-mediated regulation of E2F3, establishing a functional circ_0001741/miR-194-5p/E2F3 axis in TE2 and KYSE150 cell lines.

## Discussion

CircRNAs represent a widespread class of evolutionarily conserved non-coding RNAs with critical, yet still emerging, roles in human diseases, including cancer [23]. In ESCC, several circRNAs have been implicated in tumor progression. For instance, circ_0067934 downregulation inhibits proliferation, metastasis, and EMT, whereas CiRS-7 promotes metastasis via the miR-7/KLF4/NF-κB axis. Conversely, circ_0043898 acts as a tumor suppressor [24–26]. These findings underscore the importance of circRNAs in ESCC pathogenesis.

In our previous high-throughput sequencing of three paired ESCC tissues, we identified 573 differentially expressed circRNAs. Among these, circ_0001741 stood out due to its pronounced upregulation-shown by tissue microarray analysis to be 7.5-fold higher in ESCC tissues compared to adjacent normal tissues. Consistent with the known stability of circRNAs, circ_0001741 resisted RNase R digestion and actinomycin D treatment, confirming its covalently closed structure and potential as a stable regulatory RNA in ESCC.

Functionally, loss-of-experiments demonstrated that circ_0001741 knockdown significantly suppressed ESCC cell proliferation and invasion, indicating its oncogenic role. While multiple oncogenic circRNAs have been reported in ESCC, this study is the first to establish the functional contribution of circ_0001741 and delineate its specific molecular mechanism.

Mechanistically, we identified that circ_0001741 acts as a competitive endogenous RNA by sponging miR-194-5p, a known tumor-suppressive miRNA. This interaction was supported by the significant inverse correlation between circ_0001741 and miR-194-5p levels in clinical ESCC samples. Such a miRNA-sponging mechanism is a well-documented function for circRNAs [27], but the circ_0001741/miR-194-5p interaction itself is novel in the context of ESCC.

We further identified the transcription factor E2F3 as a direct downstream target of miR-194-5p, through which circ_0001741 exerts its oncogenic effects. E2F3 is a key regulator of cell cycle progression and has established oncogenic roles in multiple cancers [14, 28–30]. Importantly, rescue experiments confirmed that E2F3 overexpression reversed the anti-tumor effects of circ_0001741 knockdown, functionally validating this axis.

Our findings gain broader relevance when integrated with recent multi-omics insights into ESCC. For example, Zhang et al. reported co-dysregulation of RTK/RAS/PI3K and WNT/Notch pathways, alongside frequent mutations in TP53, FAT1, and NOTCH1 [31]. This contextualizes the circ_0001741/miR-194-5p/E2F3 axis within a larger network of signaling pathways that influence chemotherapy response and the tumor microenvironment (TME), particularly oxaliplatin resistance. This connection suggests that circ_0001741 may contribute to a therapy-resistant TME phenotype. Furthermore, the link between antioxidant defense and cancer pathology [32] invites future investigation into whether circ_0001741 influences ESCC progression by modulating oxidative stress pathways-a plausible direction given E2F3’s known roles in metabolism and stress responses. Additionally, Li et al. [32] provided insights into molecular determinants of combination therapy sensitivity, which could guide future studies on how circ_0001741 affects responses to cisplatin or immunotherapy in ESCC.

The field of circRNA biology continues to evolve methodologically. Recent advances, such as single-cell circRNA analysis [33], now allow precise characterization of circRNA expression across cell types. While our study used bulk tissues, applying these technologies in the future could uncover cell-type-specific roles for circ_0001741 within the heterogeneous ESCC TME. Moreover, although our work focused on circ_0001741 as a miRNA sponge, emerging evidence shows that circRNAs can also regulate transcription, bind proteins, or even encode functional peptides [23]. Thus, additional mechanisms of action for circ_0001741 cannot be excluded and warrant further investigation.

Therapeutically, targeting oncogenic circRNAs such as circ_0001741 represents a promising strategy for ESCC. Integrating insights from the cited studies not only strengthens our methodological foundation but also opens new avenues for exploring the clinical applicability of circ_0001741, especially in therapy resistance and combination treatments.

Despite these insights, our study has limitations. First, our functional data are primarily derived from in vitro models; future in vivo validation is essential to confirm the pathophysiological relevance of our findings. Second, although we established the core circ_0001741/miR-194-5p/E2F3 axis, the complete downstream transcriptional network through which E2F3 promotes ESCC progression remains to be fully elucidated. Third, the clinical correlation analysis was based on a tissue microarray; larger prospective cohort studies are needed to definitively establish the prognostic value of circ_0001741 in ESCC patients.

## Conclusions

Our results define a novel circ_0001741/miR-194-5p/E2F3 regulatory axis in ESCC, laying the groundwork for future investigation into its potential diagnostic and therapeutic applications.

## Supplementary Information

Supplementary Material 1.

Supplementary Material 2.

## Data Availability

The processed circRNA expression data supporting the key findings of this study are available from the corresponding author upon reasonable request, for academic and non-commercial use only. All other data generated or analyzed during this study are included in this published article and its supplementary information files.
