# Supplementary material for: Circ_0001741 regulates proliferation and invasion in ESCC via the miR-194-5p/E2F3 axis
Source: World J Surg Oncol. 2025 Dec 10;24:35. doi: 10.1186/s12957-025-04124-2 (PMC12801454; doi:10.1186/s12957-025-04124-2)
Supplement: Supplementary file 2 — Supplementary Material 2. [file 12957_2025_4124_MOESM2_ESM.doc]

**
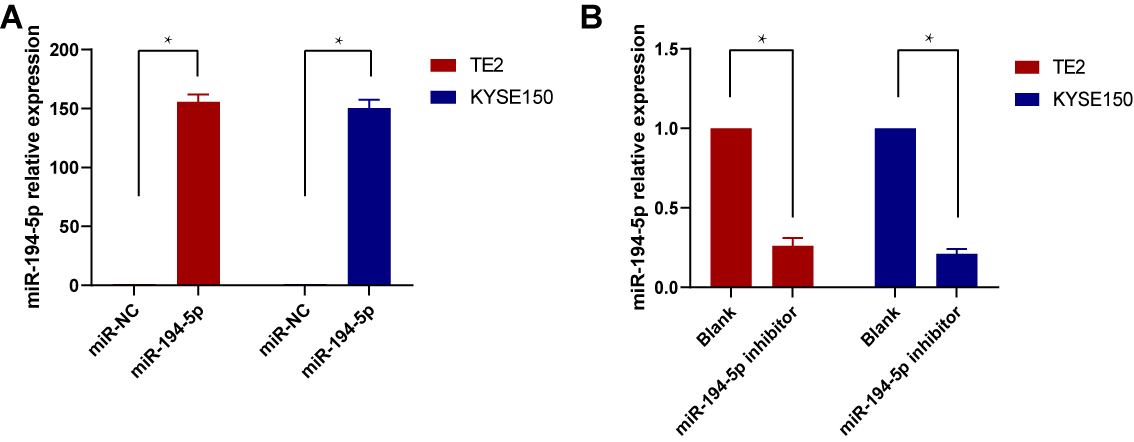
**

**Figure S1. Validation of transfection efficiency.** (A) qRT-PCR analysis of miR-194-5p expression in TE2 and KYSE150 cells after transfection with miR-194-5p mimic or negative control (miR-NC). U6 snRNA was used as an internal control. (B) qRT-PCR analysis of miR-194-5p expression after transfection with miR-194-5p inhibitor.  **P* < 0.05 vs. respective control group.

**Table S1: Primer Sequences for qRT-PCR**

| Gene Name | Sequence (5' to 3') |
| --- | --- |
| circ_0001741 | Forward: ACAGACAGGTCGAGAAGCAC |
|  | Reverse: GTGTTCCACATCCACCACCT |
| TNPO1 | Forward: CTGGAAGACGGAGAAGCTGG |
|  | Reverse: CAGGTCCATGCCTTCATTCA |
| GAPDH | Forward: GGAGCGAGATCCCTCCAAAAT |
|  | Reverse: GGCTGTTGTCATACTTCTCATGG |
| U6 | Forward: CTCGCTTCGGCAGCACA |
|  | Reverse: AACGCTTCACGAATTTGCGT |
